# Personality traits, rank attainment, and siring success throughout the lives of male chimpanzees of Gombe National Park

**DOI:** 10.7717/peerj.15083

**Published:** 2023-04-24

**Authors:** Alexander Weiss, Joseph T. Feldblum, Drew M. Altschul, David Anthony Collins, Shadrack Kamenya, Deus Mjungu, Steffen Foerster, Ian C. Gilby, Michael L. Wilson, Anne E. Pusey

**Affiliations:** 1 National Evolutionary Synthesis Center, Durham, NC, United States of America; 2 School of Philosophy, Psychology and Language Sciences, University of Edinburgh, Edinburgh, United Kingdom; 3 Wildlife Research Center, Kyoto University, Kyoto, Japan; 4 Scottish Primate Research Group, United Kingdom; 5 Department of Anthropology, University of Michigan - Ann Arbor, Ann Arbor, MI, United States of America; 6 Society of Fellows, University of Michigan - Ann Arbor, Ann Arbor, MI, United States of America; 7 Department of Evolutionary Anthropology, Duke University, Durham, NC, United States of America; 8 Mental Health Data Science, Edinburgh, United Kingdom; 9 Gombe Stream Research Centre, Jane Goodall Institute, Kigoma, Tanzania; 10 School of Human Evolution and Social Change, Arizona State University, Tempe, AZ, United States of America; 11 Institute of Human Origins, Arizona State University, Tempe, AZ, United States of America; 12 Department of Ecology, Evolution, and Behavior, University of Minnesota, St. Paul, MN, United States of America; 13 Institute on the Environment, University of Minnesota, St. Paul, MN, United States of America

**Keywords:** Chimpanzee, Personality, Fitness, Reproductive success, Life-history, Trade-offs, Gombe

## Abstract

Personality traits in many taxa correlate with fitness. Several models have been developed to try to explain how variation in these traits is maintained. One model proposes that variation persists because it is linked to trade-offs between current and future adaptive benefits. Tests of this model’s predictions, however, are scant in long-lived species. To test this model, we studied male chimpanzees living in Gombe National Park, Tanzania. We operationalized six personality traits using ratings on 19 items. We used 37 years of behavioral and genetic data to assemble (1) daily rank scores generated from submissive vocalizations and (2) records of male siring success. We tested whether the association between two personality traits, Dominance and Conscientiousness, and either rank or reproductive success, varied over the life course. Higher Dominance and lower Conscientiousness were associated with higher rank, but the size and direction of these relationships did not vary over the life course. In addition, independent of rank at the time of siring, higher Dominance and lower Conscientiousness were related to higher siring success. Again, the size and direction of these relationships did not vary over the life course. The trade-off model, therefore, may not hold in long-lived and/or slowly reproducing species. These findings also demonstrate that ratings are a valid way to measure animal personality; they are related to rank and reproductive success. These traits could therefore be used to test alternative models, including one that posits that personality variation is maintained by environmental heterogeneity, in studies of multiple chimpanzee communities.

## Introduction

Personality traits are heritable and stable behavioral or affective differences between members of a species (Gosling, 2001; Réale et al., 2007; Bell, Hankison & Laskowski, 2009; Freeman & Gosling, 2010; Dochtermann, Schwab & Sih, 2015). The discovery that personality traits are related to fitness outcomes, such as survival and reproductive success (Smith & Blumstein, 2008; Moiron, Laskowski & Niemelä, 2020), poses a puzzle because the additive genetic variation of traits under natural selection is expected to decrease to zero over successive generations, as advantageous traits become more common and disadvantageous traits disappear (Fisher, 1930).

Non-adaptive mechanisms, such as mutation-selection balance (Lande, 1975) and antagonistic pleiotropy (Roff, 1997), are unlikely to produce the observed levels of variation in personality traits (Rice, 2004; Penke, Denissen & Miller, 2007). Researchers have therefore proposed and identified several adaptive scenarios to explain the persistence of personality variation (Dingemanse & Wolf, 2010). Particularly prominent among these adaptive scenarios is that personality variation is maintained *via* life-history trade-offs (Stamps, 2007; Wolf et al., 2007; Biro & Stamps, 2008; Luttbeg & Sih, 2010). One such model finds that personality variation can be maintained because traits are linked with trade-offs between current and future reproduction (Wolf et al., 2007). In this scenario, personality traits represent alternative routes to increased fitness that could not be optimized by further selection, and the resulting set of alternate phenotypes would be akin to evolutionarily stable strategies (Maynard Smith, 1982). Critics contend, however, that trade-offs between current and future reproduction would not account for why personality variation is maintained in long-lived species, because members of such species would be able to update their strategies as their residual reproductive value changed over the life course (McElreath et al., 2007). Long-lived species thus present a crucial test of this adaptive trade-offs scenario. Nonetheless, only a few empirical studies (*e.g.*, a study of albatrosses *Diomedea exulans* by Patrick & Weimerskirch, 2015) have assessed whether personality differences are associated with trade-offs between early and late reproduction in such species (Biro & Stamps, 2008). We therefore tested whether individual differences in two personality traits were related to trade-offs in rank attainment and reproductive success in a long-lived and slowly reproducing species. To do so, we used longitudinal, prospective data on wild eastern chimpanzees (*Pan troglodytes schweinfurthii*) living in Gombe National Park, Tanzania, to examine the relationship between personality and (1) dominance rank, and (2) reproductive success over the life course.

We focused on male chimpanzees, which compete vigorously for position in a dominance hierarchy that influences reproductive success (Goodall, 1986; Muller & Mitani, 2005). Research in several chimpanzee communities reports that high-ranking males sire a disproportionate share of offspring (Boesch et al., 2006; Wroblewski et al., 2009; Gilby et al., 2013; Surbeck et al., 2017). While female dominance rank is also associated with reproductive success (Pusey, Williams & Goodall, 1997), female dominance rank in Gombe appears to depend little on daily competitive interactions. Instead, females have a queueing system whereby they start at a given rank based on some unknown aspect of competitive ability (perhaps body size), and thereafter change rank only slowly as females die, mature, and transfer among communities (Foerster et al., 2016). Male dominance rank thus changes more dynamically, and seems more likely influenced by variation in personality traits. We therefore focused our study on data from male chimpanzees.

When competing for status, male chimpanzees use a mix of aggressive and cooperative tactics. The highest-ranking males tend to have the highest rates of aggression towards other males (Muller & Mitani, 2005), while lower-ranking males may groom higher-ranking males in exchange for tolerance, support, or mating concessions (Duffy, Wrangham & Silk, 2007; Bray, Pusey & Gilby, 2016; Feldblum et al., 2021). Other males form aggressive coalitions and social bonds, which appear to facilitate subsequent rises in rank (Nishida, 1983; Gilby et al., 2013; Watts, 2018; Bray, Feldblum & Gilby, 2021). Finally, there is anecdotal evidence for other tactics that males may use to attain and maintain high rank despite lower resource holding potential. These include “allegiance fickleness”, or strategically changing alliance partners (Nishida, 1983), the strategic use of grooming (Foster et al., 2009), and the use of tools—including, in one exceptional case, empty kerosene cans—to make displays more intimidating than they otherwise would be (Goodall, 1988).

Still, dominance rank is far from the sole determinant of male reproductive success. Considerable variation also exists in male chimpanzee reproductive tactics and outcomes. Male chimpanzees that are more aggressive towards females mate with and sire more offspring with those females (Muller et al., 2011; Feldblum et al., 2014; Kaburu & Newton-Fisher, 2015; Reddy et al., 2021). Alternatively, males may improve their mating success by grooming females (Kaburu & Newton-Fisher, 2015; Reddy et al., 2021). In addition, males form aggressive coalitions with other males that can provide short-term access to mating opportunities (Watts, 1998). Finally, lower-ranking males have higher mating and siring success when forming social bonds with and providing coalitionary support to the alpha male (Duffy, Wrangham & Silk, 2007; Bray, Pusey & Gilby, 2016; Feldblum et al., 2021), as well as when they form large networks of strong social bonds with other subordinate males (Feldblum et al., 2021).

The diverse behavioral tactics males use when competing for status and reproductive opportunities, as well as the long-term stability of some behavioral phenotypes in chimpanzees (Tkaczynski et al., 2020), suggest that personality traits could influence male competitive behavior and, in turn, fitness outcomes. Little is known about the influence of personality traits on fitness outcomes over the lifespan of wild chimpanzees. Previous studies have identified relationships between personality and rank in several taxa, including male rainbowfish *Melanotaenia duboulayi* (Colléter & Brown, 2011), spotted hyenas *Crocuta crocuta* (Gosling, 1998), and Barbary macaques *Macaca sylvanus* (Konečná et al., 2012), and between personality and leadership in humans (Judge et al., 2002). These studies, however, have been cross-sectional or involved measures taken at two time points. Therefore, these studies cannot determine whether different personality traits benefit individuals at different points in their lives.

Other studies report inconsistent relationships between personality traits and reproductive success in long-lived primates. Among common marmosets *Callithrix jacchus*, Masilkova et al. (2022) reported that females with higher Agreeableness and Inquisitiveness had shorter interbirth intervals and higher fecundity, respectively, and that higher Conscientiousness (another trait) in males was associated with increased likelihood of producing twins rather than triplets. On the other hand, Brent et al. (2014) reported no associations between personality traits and reproductive success in rhesus macaques *Macaca mulatta*. In humans, the relationship between personality traits and reproductive success differs across studies and often by sex and social factors. In men and women living in the United States, higher Extraversion, lower Conscientiousness, and lower Openness predicted having more children and grandchildren, and higher Agreeableness predicted having more grandchildren (Berg et al., 2014). In a traditional community in rural Senegal, higher Extraversion predicted greater reproductive success in men, and Neuroticism predicted greater reproductive success in women, although among women who belonged to a low social class, Neuroticism was associated with poorer quality offspring (Alvergne, Jokela & Lummaa, 2010). In another such study of a traditional society, relationships between personality and fertility differed by region among the Tsimané (Gurven et al., 2014). However, because these studies do not always include longitudinal data, it is unclear whether some inconsistency across these studies may result from different traits being associated with reproductive success at different stages of the life course.

Based on these findings, we tested (1) whether personality was associated with either rank or siring success among adult male chimpanzees, and (2) whether associations between personality and rank or siring success varied over the lifespan of these males. We generated behavioral measures of dominance rank from submissive ‘pant-grunt’ vocalizations (Marler, 1976) and measures of reproductive success from 55 siring events between 1980 and 2014. Our personality measures were based on ratings made in 2010 by long-term Tanzanian field researchers (Weiss et al., 2017).

For the present study, we used data on six chimpanzee personality traits identified by King & Figueredo’s (1997) factor analysis of ratings made on 100 zoo-housed chimpanzees. The traits are: Dominance, which characterizes individuals in terms of their competitive prowess, fearlessness, and boldness; Extraversion, which describes individuals in terms of their sociability, positive emotions, and activity; Conscientiousness, which describes the degree to which individuals are predictable, tame, and focused; Agreeableness, which captures tendencies to be pro-social, for example being helpful and gentle; Neuroticism, which describes levels of anxiety, emotional instability, and negative affect; and Openness, which characterizes individuals in terms of their curiosity, novelty seeking behavior, and the degree to which they explore their physical and social environments.

There is considerable evidence that the traits identified by King & Figueredo (1997) used here summarize the major features of chimpanzee personality. First, these traits do not appear to be products of the environment in which they were rated or the identity of the researchers who rated the chimpanzees. The same or similar traits were found in chimpanzees living in naturalistic sanctuaries in the Republic of the Congo (Conkouati Sanctuary; King, Weiss & Farmer, 2005) and in both Zaire and Sierra Leone (Chimfunshi Wildlife Orphanage Trust and the Tacugama Chimpanzee Sanctuary, respectively; Ortín et al., 2019), chimpanzees living in Yerkes National Primate Research Center (Weiss, King & Hopkins, 2007), and chimpanzees living in Japanese zoos, research centers, and a sanctuary (Weiss et al., 2009). Second, these traits do not appear to be products of how questionnaires were developed. Studies of chimpanzees that have used personality questionnaires derived using different methods identified similar personality traits (Freeman et al., 2013). Third, similar traits were identified by a principal components analysis of naturally occurring social behaviors in captive chimpanzees (Van Hooff, 1970). Finally, these traits correspond to traits measured using behavioral tests in studies of other species (Table 1).

For this study, we focused on two traits: Dominance and Conscientiousness. As noted above, among human males, Extraversion is related consistently to greater reproductive success, including in traditional (Alvergne, Jokela & Lummaa, 2010; Gurven et al., 2014) and modern societies (Berg et al., 2014), and both boldness and aggression are related to reproductive success in nonhuman species (see Smith & Blumstein, 2008 for a review). Chimpanzee Dominance includes traits related to assertiveness, a facet of Extraversion in humans (Costa & McCrae, 1995), and both boldness and aggression (*e.g.*, King & Figueredo, 1997).

The behavioral measure “dominance rank” and the personality trait “Dominance” are semantically similar and may appear interchangeable, but they describe different constructs that are derived independently using distinct data. The behavioral measure, dominance rank, describes an aspect of dyadic relationships. An individual can be said to be dominant to another if they consistently win competitive interactions. Dominance rank is a narrow construct that reflects position in a dominance hierarchy that emerges from patterns of dyadic dominance relationships (Hinde, 1978; Bernstein, 1981; Watts, 2010). On the other hand, the personality trait Dominance is a broad multivariate latent construct. This construct describes cross-context, consistent, and heritable individual differences in behavioral and affective disposition associated with individual quality or competitive prowess (Maslow, 1937; King & Figueredo, 1997; Weiss, King & Figueredo, 2000; Wilson et al., 2017). Chimpanzees higher in Dominance are not only perceived as more dominant and less submissive, but also as more independent, decisive, intelligent, persistent, bullying, stingy, and manipulative, and less dependent, fearful, timid, cautious, vulnerable, and anxious (King & Figueredo, 1997; Weiss et al., 2009). In chimpanzees, the personality trait labeled “Conscientiousness” describes the degree to which individuals are predictable in their behavior and reactions, organized, goal directed, and non-aggressive, or tame (King & Figueredo, 1997; Weiss et al., 2009; King & Weiss, 2011). Dominance and Conscientiousness are also distinct from dominance rank in that none of the questionnaire items used to measure these traits asks about receiving pant-grunts or the probability of winning aggressive interactions.

**Table 1:** Personality variables, definitions, and related traits.

**Variable**	**Chimpanzee described as...**	**Animal personality trait(s)**
Dominance	Dominant, decisive, *not* dependent	Boldness *and* aggressiveness (Réale et al., 2007)
Extraversion	Sociable, active, *not* individualistic, *not* solitary	Sociability *and* activity (Réale et al., 2007)
Conscientiousness	Predictable, not impulsive, *not* reckless	Self-control (MacLean et al., 2014); predictability (Biro & Adriaenssens, 2013)
Agreeableness	Sympathetic, helpful, sensitive	Cooperativeness (Dantzer et al., 2019)
Neuroticism	Excitable, *not* stable	Vigilance (Brent et al., 2014)
Openness	Inventive, inquisitive, curious, innovative	Exploration (Réale et al., 2007)

We first conducted preliminary analyses in which we validated these rating-based measures by examining their relationships with single components of rank trajectories. We then conducted our primary analyses in which we examined the relationships between (1) lifetime dominance rank trajectory, and (2) siring success, and the personality traits Dominance and Conscientiousness, assessed once in 2010. If alternative traits (*e.g.*, high and low Dominance) are associated with higher rank scores or reproductive success at different stages in the life course, then this would support the hypothesis that variation in personality traits can be maintained by trade-offs between current and future fitness (Stamps, 2007; Wolf et al., 2007; Biro & Stamps, 2008). Alternatively, if personality is associated with rank or reproductive success but this association remains static over the life course, this would not support the trade-offs scenario, or suggest that it cannot explain the persistence of trait variation in long-lived species (McElreath et al., 2007).

## Materials & Methods

### Study site and subjects

Researchers have collected data during daily focal follows (Altmann, 1974) on adult chimpanzees in the Kasekela community of Gombe National Park, Tanzania since 1973. During these follows, pairs of researchers record party composition *via* scan samples at 15-minute intervals, as well as the timing of all individual arrivals and departures from the focal individual’s ranging party. In addition, researchers record all-occurrences data on aggression, other dominant and submissive behaviors, mating, hunting, and other behaviors in narrative notes. Finally, researchers record the focal subject’s location every 15 min on paper maps of the park (Goodall, 1986; Wilson, 2012) and, since 2018, with portable GPS units. The research team has conducted a mean of 297 (SD = 58) days of observation each year since 1973.

Subjects included 34 males. We included males that survived until at least 12 years of age, and that survived beyond the start of 1978 when dominance rank data are available (see below). Males begin to travel with adult males and challenge other males for position in the hierarchy around age 12 (Pusey, 1990). The youngest age of first reproduction among males at Gombe was 12.0 years, or 11.4 on the date of siring (Foster et al., 2009; Feldblum et al., 2021).

## Measures

### Dominance rank

To operationalize dominance rank, we used submissive pant-grunt vocalizations with an unambiguous actor and recipient to generate measures of male dominance rank where both actor and recipient were at least 12 years old. This is a well-established and standard method of estimating dominance rank in chimpanzees because pant-grunts are only given from the subordinate to the dominant in any pair (Marler, 1976; Goodall, 1986). The data set included 6,153 total dyadic pant-grunt events (range = 57 to 2,082 per chimpanzee) from 1978 to 2015.

To generate a dynamic longitudinal record of dominance rank, we used a maximum likelihood implementation of the Elo rating method (Elo, 1978; Foerster et al., 2016) in the package “EloOptimized” in R version 4.1.1 (Feldblum, Foerster & Franz, 2019; R Core Team, 2020). To ensure that we accurately captured relationships between personality and rank among young males, we estimated both initial Elo scores for each male upon entry into the hierarchy, as well as the scaling parameter *k* (Model 3 in Foerster et al., 2016). Because absolute Elo scores could be influenced by group composition, we transformed Elo scores to represent, for each individual on each day, the sum of the pairwise likelihoods of winning an agonistic encounter against all other males in the hierarchy standardized by the total number of individuals present in the community (see Foerster et al., 2016 for details). This measure varies between 0 and 1 while preserving the daily distribution of Elo scores. This resulted in 626 to 11,198 daily observations of Elo score per individual (median = 5,255.5), totaling 149,005 observations.

### Personality trait ratings

A discussion of the advantages and disadvantages of using ratings and other measures, including behavioral measures, were the subjects of a target article by Uher (2008a), the Open Peer Commentary and Uher’s 2008b reply, a review by Freeman & Gosling (2010), and a chapter by Weiss & Adams (2013). These sources concurred that ratings of nonhuman primates are reliable and valid, and so produce useful information. This is not surprising for the evidence is considerable. The interrater reliabilities of ratings of chimpanzee personality traits (Crawford, 1938; Buirski, Plutchik & Kellerman, 1978; King & Figueredo, 1997; King, Weiss & Farmer, 2005; Weiss, King & Hopkins, 2007; Dutton, 2008; Uher & Asendorpf, 2008; Weiss et al., 2009; Freeman et al., 2013) are comparable to the interrater reliabilities of ratings of human personality traits (Mõttus et al., 2017). Moreover, studies that use ratings have revealed evidence for mean-level changes in chimpanzee personality traits, some of which resemble those found in humans, and evidence for the stability (the retest reliability) of these traits (Dutton, 2008; King, Weiss & Sisco, 2008; Uher & Asendorpf, 2008; Rawlings et al., 2020). There is also evidence that personality ratings measure behavioral, affective, and cognitive characteristics of chimpanzees. Personality traits measured using ratings are prospectively related to measured behavior (Buirski, Plutchik & Kellerman, 1978; Pederson, King & Landau, 2005; Vazire et al., 2007; Uher & Asendorpf, 2008; Freeman et al., 2013; Brosnan et al., 2015) and heritable (Weiss, King & Figueredo, 2000; Latzman et al., 2015; Wilson et al., 2017). Moreover, higher Dominance and Conscientiousness are positively associated with percentage of gray matter in the subgenual cingulate cortex (Blatchley & Hopkins, 2010), Agreeableness is associated with longevity in males (Altschul et al., 2018), and higher Conscientiousness, Openness, and Dominance have been associated with better performance on cognitive tasks (Hopper et al., 2013; Altschul et al., 2017; Padrell et al., 2020). Finally, rating-derived traits are not the products of anthropomorphic projections (Weiss et al., 2012).

For this study, we computed chimpanzees’ scores on six personality traits using ratings data collected in 2010 (https://osf.io/s7d9d/). Weiss et al. (2017) detail how these data were collected and on the reliability, stability, and validity of all six traits. Briefly, 18 Tanzanian researchers provided 494 ratings for 141 chimpanzees that they followed during their tenure (up to 35 years; see Weiss et al., 2017 for details). Each of these researchers completed ratings for between 21 and 43 chimpanzees on a 24-item Swahili-language version of the Hominoid Personality Questionnaire (Weiss et al., 2009; Weiss et al., 2017; Weiss, 2017). Each questionnaire item comprises an adjective and a brief description that sets the adjective in the context of behavior. For example, the item “sociable” was “**SOCIABLE**: Subject seeks and enjoys the company of other chimpanzees and engages in amicable, affable, interactions with them.” (boldface in original). The questionnaire instructs raters to assign each item a score from 1 (*Displays either total absence or negligible amounts of the trait*) to 7 (*Displays extremely large amounts of the trait*) based on the “... chimpanzee’s own behaviors and interactions with other chimpanzees” (King & Figueredo, 1997). The questionnaire also instructs raters to not discuss their ratings with one another. These items were used to calculate a score for each of the six traits (see Text S1: Personality Trait Calculations in Weiss et al., 2017). It is important to note that, although rater impressions originated from observations of how individuals behave and how others behave towards those individuals, these impressions were based on a range of contexts. As such, items and trait scores capture behavioral, affective, and other tendencies that are consistent across many contexts.

In the full sample of 106 chimpanzees in the Kasekela community that were rated by at least two research assistants, 19 of the 24 questionnaire items showed evidence of adequate levels of interrater reliability for further analysis (Weiss et al., 2017). The interrater reliabilities of the mean scores (Shrout & Fleiss, 1979) suggested that 11–66% (median = 38%) of the variation between chimpanzees in these 19 traits was attributable to the chimpanzees as opposed to sources of non-systematic variance, that is, error variance (Weiss et al., 2017). These 19 items were used to create six trait scores for the 106 chimpanzees from the Kasekela community (Weiss et al., 2017). These scores were based on previous chimpanzee personality research (King & Figueredo, 1997; Weiss et al., 2009). The interrater reliabilities of these trait scores based on the average of raters’ ratings were 0.51 for Dominance, 0.40 for Conscientiousness, and 0.39, 0.40, 0.35, and 0.53, for Extraversion, Agreeableness, Neuroticism, and Openness, respectively (see Table 3 in Weiss et al., 2017). Thus, around 40–50% of the variance in these scores was attributable to the chimpanzees with the remaining variance being non-systematic. These interrater reliabilities are comparable to repeatabilities (Boake, 1989; Hayes & Jenkins, 1997) of behavioral measures of personality traits across multiple taxa, which Bell, Hankison & Laskowski (2009) reported to be 0.37 (p. 774). Thus, just under two-fifths of the variance in measures, such as behavioral observations or responses to behavioral tests, such as the novel-object test were attributable to between-animal effects; the remaining three-fifths of the variance was error variance.

There is evidence for the long-time stability (retest reliability) of personality ratings among chimpanzees in the Kasekela community. In 1973, seven students and post-doctoral researchers rated 24 members of this community using the Emotions Profile Index (Buirski, Plutchik & Kellerman, 1978; Buirski & Plutchik, 1991). An examination in these chimpanzees of the correlations between the eight traits measured by the Emotions Profile Index and the six traits examined in this study found that the traits from these scales were significantly correlated in ways consistent with the meanings of the traits, *e.g.*, the Emotions Profile Index trait “Trustful” only had a significant (positive) correlation with “Agreeableness” (Weiss et al., 2017).

Ratings data were collected on 106 chimpanzees from the Kasekela community in 2010, and 28 males from that sample served as subjects in the present study. Twelve researchers completed 88 questionnaires for these males: 25 males were rated by three of these researchers, two were rated by four, and one was rated by five. The researchers knew the chimpanzees that they rated for between 2 and 29 years (mean = 15.7, SD = 5.8). When the researchers began to collect behavioral data on the 28 subjects, the chimpanzees ranged in age from less than one year old to 26 years old (mean = 11.3, SD = 6.3). When the researchers last saw the 28 males, the chimpanzees ranged in age from 9 to 41 years (mean = 27.0, SD = 8.8).

## Analyses

Prior to fitting models, we conducted preliminary analyses to validate the personality ratings data. For all of our analyses, to facilitate comparison of effect sizes, we *z*-transformed personality trait scores, male age, and male Elo scores. All of the analyses focused on Dominance and Conscientiousness, which we expected would be important given the literature on male dominance rank (Goodall, 1986; Muller & Mitani, 2005). However, for our primary analyses, we also included Extraversion, Agreeableness, Neuroticism, and Openness when adding these traits was supported in model comparisons. We conducted these tests of the other traits because, by including them, we reduced both the likelihood that our parameters would be biased (Wilms et al., 2021) and the likelihood of misclassifying personality traits (Carter et al., 2012b; Carter et al., 2012c; Carter et al., 2012a).

### Preliminary analyses: validating personality data

We first tested for the validity of Dominance and Conscientiousness by using bivariate regressions to examine their associations with elements of dominance rank trajectories. These tests were based on restricted sample sizes created using arbitrary criteria to include/exclude subjects that were appropriate to each measure (*e.g.*, minimum age of survival for some analyses).

We delineated six elements of dominance rank trajectories, defined as follows: (1) Elo score at entry (restricted to the 21 males that entered the hierarchy at age 12); (2) slope of initial rise in Elo score (change in Elo score between entry and the first date of achievement of highest ordinal rank, divided by the time between entry and first achievement of highest ordinal rank; restricted to the 21 males that entered the hierarchy at age 12); (3) highest Elo score achieved (restricted to the 19 males for which we have rank data until at least age 26); (4) highest ordinal rank achieved (restricted to the 19 males for which we have rank data until at least age 26); (5) length of time at high Elo (the time difference between the last day of highest ordinal rank achieved and the first day of the same; restricted to the 11 males that rose to at least an ordinal rank of 3; (cf. Gilby et al., 2013) entered the hierarchy before age 16, and for which we have rank data until at least age 26); and (6) slope of rank decline (change in cardinal Elo score over two years following the last date of highest ordinal rank, which was restricted to 10 males that survived at least two years beyond their last recorded date at their highest ordinal rank).

We re-ran these analyses excluding Pax, a male that sustained severe testicular injuries as a juvenile and remained low ranking and peripheral throughout his life (Goodall, 1986). These additional analyses were conducted to ensure that his unusual rank trajectory did not bias results of these analyses.

### Study 1: personality and lifetime rank trajectories

We sought to avoid problems related to sample size and inclusion criteria. We therefore used generalized additive mixed models (GAMMs; Wood, 2006) with daily Elo scores for all 28 males that were at least 12 years of age, and for whom we had personality data and rank data between 1978 and 2015. Because GAMMs are flexible enough to accommodate a wide range of trajectories, we did not exclude Pax for these analyses. These males were thus present for a mean of 14.1 years beyond the age of 12 (range 1.7–30.7 years).

In our GAMMs, we examined associations of age, personality, and cardinal Elo scores. These models were implemented in the R package mgcv (Wood, 2011). We chose to use GAMMs because unlike linear models, additive models are suited to modeling non-linear trajectories, such as male chimpanzee rank (Foerster et al., 2016). In our models, we used all Elo outcome data available (149,031 observations).

Different types of splines must be used for fixed and random effects. For fixed effects, we used tensor product interaction (ti) smooths because they allow separation of main and interaction effects as both can be specified using distinct ti formula terms. We opted to use penalized cubic regression splines with shrinkage for these main effects and interactions as these splines are more conservative and can shrink a parameter estimate to zero, thus removing it from the equation.

We fit six models. The first included only the main effect of age. The second and third added *z*-transformed Dominance and Conscientiousness, respectively, along with their interaction with age. The fourth included both Dominance and Conscientiousness, and their interactions with age. The fifth included all six chimpanzee personality dimensions and their interactions with age. The sixth was added at the request of a reviewer, to deal with potential biases of autocorrelation and includes the same fixed effects as model five, but an additional random effect—see below. For random effects, to reduce overfitting, we specified smooths with ridge penalties for each individual variable. The model included random intercepts and random slopes for each age and personality main effect, with random effect grouping determined at the level of individual chimpanzees, and a random effect for date in the sixth model.

Additive models do not produce standard regression coefficients. Smoothing splines parsimoniously fit curves, or surfaces in the case of interactions, to the data. The effect of every predictor variable can be shrunk to a linear relationship, or even to zero, if doing so generates optimal fit, minimizing overfitting. Shrinking the effect of a variable to zero is a special case where the model suggests that there is no relationship between the predictor and outcome variables, and the variable can effectively be removed from the regression equation. Therefore, although overall significance for each spline can be assessed through quantitative statistics, the effect of variables must be interpreted graphically by plotting the splines. We determined the approximate overall significance as well as specific regions of significance for two-dimensional interaction surfaces to understand the relationships among age, personality, and rank.

The modeling approach and concomitant fit statistics in GAMM are not equivalent to those of linear models. Therefore, although AIC statistics can be obtained from GAMM models, they are not reliable in the same way as are the fit statistics derived from linear models. This is because GAMM AICs cannot be scaled appropriately for weighting, so the model with the best fit always looks like it fits the data perfectly, which is not accurate. We therefore assessed model fit with deviance explained, adjusted *R*^2^, and robust cross-validation measures (see Validation Analyses section in Text S1). Where appropriate, models were compared using *χ*^2^ tests of twice the difference in minimized smoothing parameter selection score (in this case restricted maximum likelihood). The section of the plots that correspond to individuals in their 30s and 40s are based on fewer individuals because fewer males survive to these ages. These sections of the surfaces are thus not as reliable, and so we exercised caution when interpreting them. For more on how to fit and interpret GAMMs, we recommend Wood (2006).

### Study 2: personality and reproductive success

For the second study, we analyzed siring events with known paternity between 1986 and 2014, using personality and other traits to predict likelihood of male siring success. We included males as potential sires if they were at least 11 years old on the siring date, as the youngest male known to sire an offspring in Gombe was 11.4 years old at the time of siring (and 12.0 years old at birth, as noted above), and we had both genetic and personality data for the males. This resulted in a data set that included 55 siring events, with 24 unique mothers and 22 unique males. These males had a mean age of 22.7 (range 11 to 40.8) years, and were present in the data set for a mean of 27 (range 1–55) siring events. The dataset had a median 11 (range 8–14) potential sires per siring event (see Inclusion Criteria in Text S1).

For each siring event, we estimated siring date by subtracting the Gombe-specific mean gestation length for singleton births of 228 days (Feldblum et al., 2021) from each offspring’s date of birth. We then recorded each potential sire’s cardinal Elo score and age on each siring date. Because closely related male–female dyads are less likely to mate and reproduce in this population (Feldblum et al., 2014; Walker et al., 2017), we also included pairwise genetic relatedness values (R; Queller & Goodnight, 1989) for each potential male sire with each mother in the sample. Genetic relatedness data were missing for some dyads because genetic sampling began after the death of some subjects. We excluded those dyads from the analysis (see above). To quantify male reproductive success, we used paternity data based on genetic material collected from non-invasive fecal sampling using a microsatellite-based exclusion method (Wroblewski et al., 2009; Walker et al., 2017). All paternities in the current analysis were reported in earlier publications (Constable et al., 2001; Wroblewski et al., 2009; Gilby et al., 2013; Feldblum et al., 2014; Walker et al., 2017).

For the analyses, we used information-theoretical model comparisons (Burnham & Anderson, 2002) to determine (1) whether Dominance and Conscientiousness were important predictors of male siring success controlling for dominance rank score, and (2) whether different levels of these personality traits were associated with male siring success at different times in the life course.

Following Feldblum et al. (2021), we first constructed a base model against which to compare to more complex models. The base model was a binomial generalized linear mixed model with siring success as a binary outcome variable and a logit link function. The base model included male age, male Elo score on the estimated date of siring, and male relatedness to the mother as predictor terms, as well as random intercepts for male identity. We then constructed additional models to compare with the base model to determine if including Dominance and Conscientiousness, as well as interactions between personality trait scores and other predictor terms such as Elo score and age, would improve fit. We also included models testing potential nonlinear longitudinal relationships between personality and male siring success; this was to test the prediction that alternative personality traits (*e.g.*, high and low Dominance) should be associated with higher reproductive success at different stages in the life course. These models included both linear and squared personality trait scores, and interactions between those terms and male age. Additionally, as with the analysis of male Elo scores from Study 1, we included a model that included all six traits to reduce the likelihood of omitted variable bias and incorrectly classifying personality traits.

We compared model fits using corrected Akaike’s Information Criterion (AICc) scores (Burnham & Anderson, 2002). Models with lower AICc scores are more likely to minimize information loss when predicting siring success. We also calculated Akaike weights for each model, which can be interpreted as conditional probabilities for each model (Burnham & Anderson, 2002). All models were fitted using the R package lme4, version 1.1–27.1 (Bates et al., 2014). Model comparisons were conducted using the MuMIn package, version 1.43.17 (Bartoń, 2015).

## Ethical Approval

Approvals for the long term study, and data collection by AW (2010-296-NA-2009-123), were granted by the Tanzania Commission for Science and Technology, the Tanzania Wildlife Research Institute, and Tanzania National Parks.

## Results

### Preliminary analyses: validating personality ratings data

Dominance and Conscientiousness were inversely correlated in the full sample of 46 males for whom personality ratings were available (*r* =  − 0.596, *t* =  − 4.918, *df* = 44, *p* < 0.001). Due to the small sample sizes in the validity analyses, we report below associations where effect size and confidence intervals suggest an association may be present even if the 95% confidence interval does not exclude 0. Note that cardinal Elo scores can range from 0 to 1. Dominance was positively associated with Elo score at entry (*β* = 0.027, 95% confidence interval (95% CI) = [−0.016–0.071]). This represents about 7% of the total range of Elo scores at entry (which ranged from 0.08 to 0.48). This association remained after excluding Pax (*β* = 0.036, 95% CI [−0.009–0.080]). Dominance was also positively associated with the slope of initial Elo rise (*β* = 0.020, 95% CI [0.005–0.036]). It was not possible to include Pax in this analysis because he never increased in ordinal rank after entering the hierarchy. Among the 19 males that survived until at least age 26, Dominance was positively associated with the highest cardinal Elo score achieved (*β* = 0.099, CI [0.008–0.190]) and highest ordinal rank achieved (*β* = −0.986, 95% CI [−1.934 to −0.038]), although these effects were attenuated after excluding Pax (cardinal Elo score: *β* = 0.052, 95% CI [−0.030–0.134]; ordinal rank: *β* = −0.359, 95% CI [−1.034–0.317]).

Although a smaller effect, there was evidence that Conscientiousness was inversely associated with Elo score at entry (*β* = −0.035, 95% CI [−0.082–0.011]). This association remained after excluding Pax (Elo score at entry: *β* = −0.039, 95% CI [−0.086–0.008]). Slope of initial Elo rise was also inversely associated with Conscientiousness (*β* = −0.013, 95% CI [−0.031–0.006]). As before, Pax was not included in the latter analysis because his Elo score never rose beyond his score at entry. Finally, among the 12 males (excluding Pax) for whom we have Elo score data by age 16, and until at least age 26, and that rose to at least the third position in the dominance hierarchy, there was an inverse association between time at their highest ordinal rank and Conscientiousness: males that were lower in this trait had longer tenures at the highest ordinal rank they achieved (*β* = −0.223, 95% CI [−0.393 to −0.054]).

The remaining components of rank trajectories were not associated with Dominance or Conscientiousness. This may reflect the fact that calculating measures of these latter components required further restricting sample sizes and choosing arbitrary age cutoffs for inclusion.

### Study 1: personality and lifetime rank trajectories

**Figure 1:**
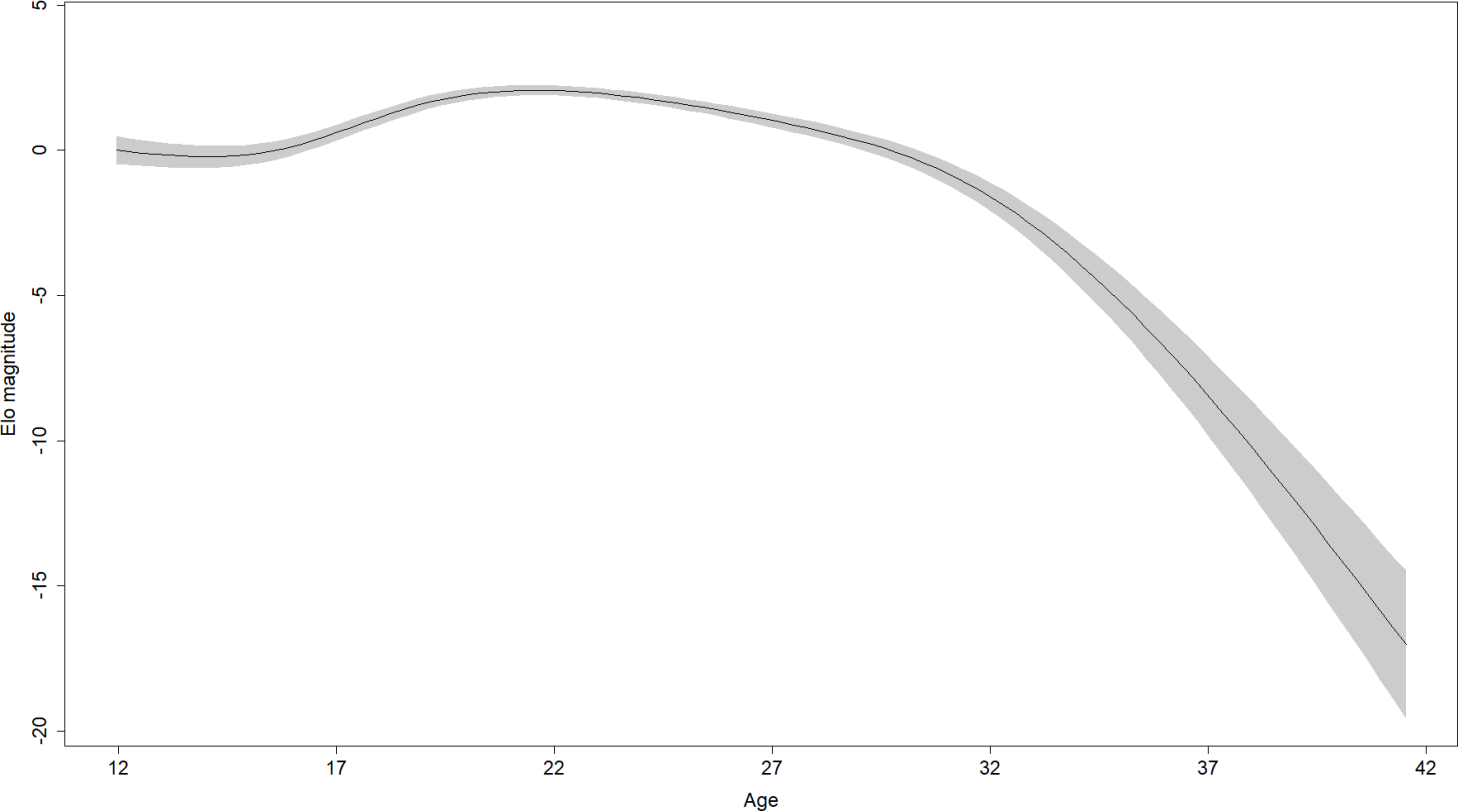
Regression spline of Elo rank score associated with different ages. The black line indicates the spline and the shaded area is the 95% confidence region (±2 standard errors). Elo magnitude indicates rank position relative to others’ rank.

**Figure 2:**
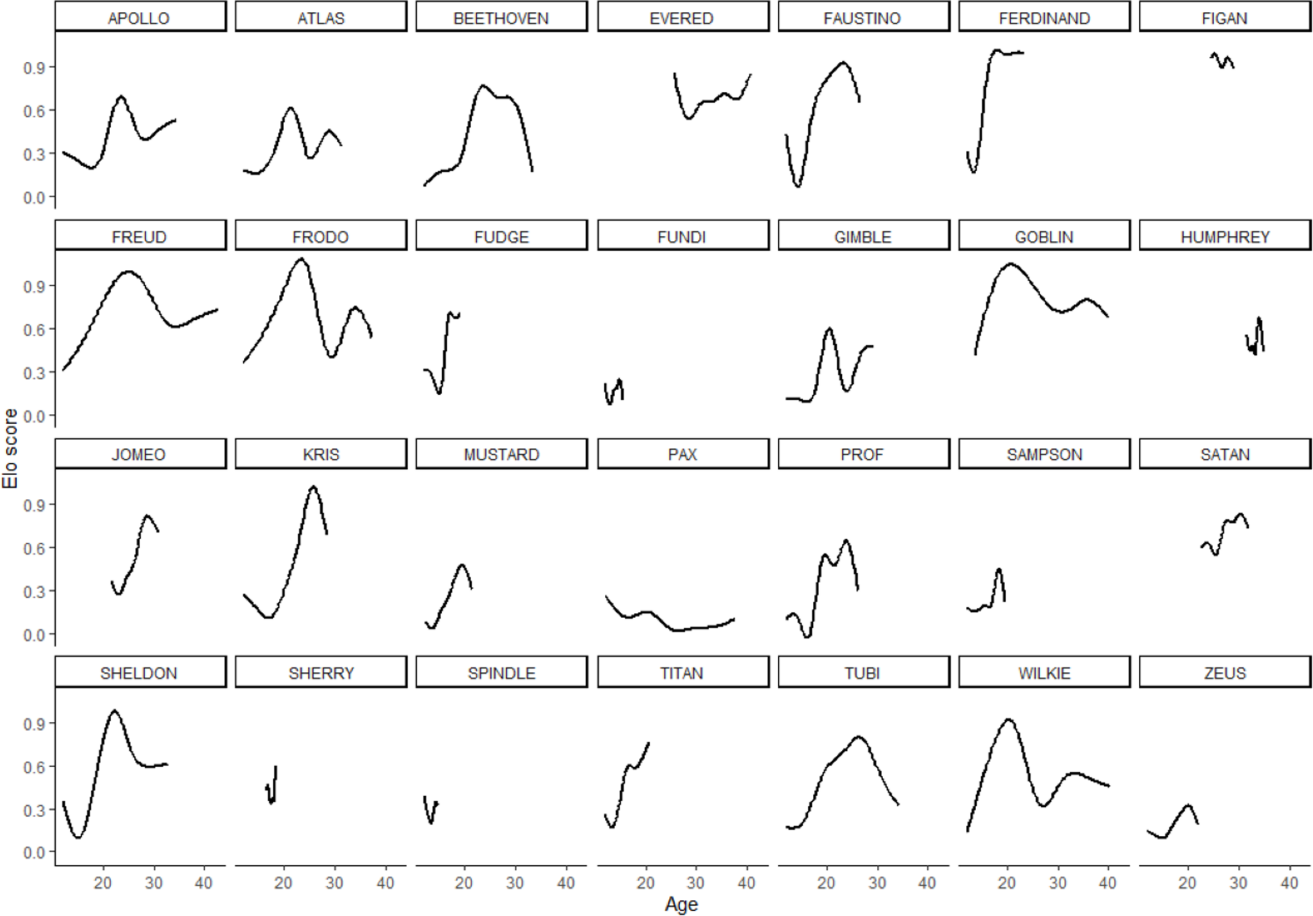
Rank score trajectories of all males in the sample. Trajectories for each individual are additive model regression splines, smoothed using the minimum, parsimonious degrees of freedom.

In the 28 males in the sample, rank scores tended to rise gradually, experience a peak, and then fall gradually (Fig. 1). Yet the shape of rank score trajectories still varied considerably between individuals: some individuals changed dramatically over short intervals, other individuals never achieved marked gains in rank score, and the trajectories of others were cut short by death or because the study period ended while these individuals were young (Fig. 2).

To test whether Dominance or Conscientiousness play different roles in rank attainment at different times in a male chimpanzee’s life, we modeled overall and time-varying relationships between individual personality traits and rank. GAMM fit was strong: for the simplest age model, 82% of the variance (adjusted *R*^2^) and 78% of the deviance (the likelihood-based goodness-of-fit statistic *D* = 12905.53) in rank scores could be explained by the model. The fifth model, which contained all six personality dimensions and their interactions with age, fit the data well: 91% of the variance (adjusted) and 89% of the deviance (*D* = 6342.319) in rank scores could be explained (*χ*^2^ = 140617.6, effective *df* = 28, *p* < 0.001). At a reviewer’s request, we fit a more complex model with the addition of a random effect for date. This more complex model (Table S1, Figs. S1–S5) accounted for 91% of the variance (adjusted *R*^2^) and 89% of the deviance (*D* = 6375.037), but it differed in no substantive way from the simpler model, so subsequent analyses interpreted the less complex version of the GAMMs, the fifth model.

We also used cross-validation, which compares real outcome values for data not used to fit the model with what the model predicts the outcome values will be when those data are fed to the model as new inputs. We evaluated the cross-validation performance of the fifth model using the mean squared error, which represents the difference between true values and model predicted values. If the model’s prediction was perfect, the mean squared error would equal zero. Cross-validation yielded strong mean squared errors of 0.0078 and 0.0449 for stratified 10-fold and forward chaining average predictive error, respectively. In general, scores seemed to be poorest in the middle, where the training dataset included between six and eight folds. On average, scores were good and compared favorably with the test error of the models (Table S2). While the results of 10-fold cross-validation do not rule out overfitting, they do suggest that the scores are inflated due to temporal autocorrelation, as expected. We also cross-validated the sixth model, and its critical forward chaining score was 0.0452 (Table S3), indicating no improvement on the performance of the simpler fifth model. We also checked for multicollinearity by calculating variance inflation factors (Fox & Monette, 1992) for our best-fitting model and our base model using the car package, version 3.0-11 (Fox & Weisberg, 2019). All variance inflation factors were below 4, indicating no risk of multicollinearity.

**Figure 3:**
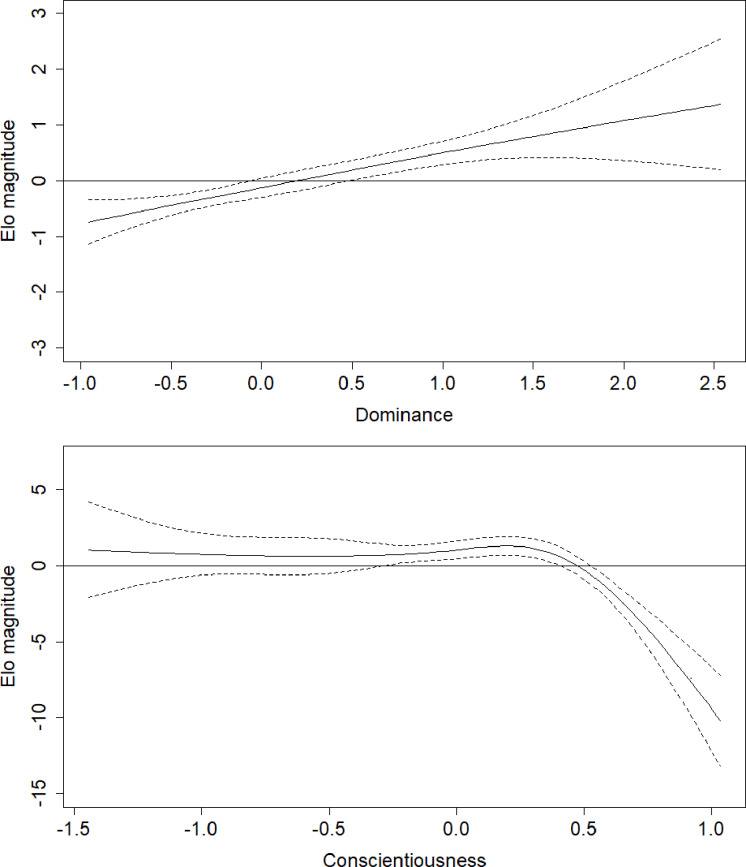
Regression splines of Elo rank score associated with Dominance and Conscientiousness. The solid black line indicates the spline, dashed lines indicate the bounds of the 95% confidence region (±2 standard errors). The *x* axes show *z*-transformed personality trait scores. Elo magnitude indicates rank position relative to others’ rank.

Parameter estimates for the fifth model are presented in Table 2. Effects in generalized additive models are represented by effective degrees of freedom (*edf*), which represent the shape of the association between the independent variables and their interactions, and the dependent variable. An *edf* of 1 indicates a linear relationship, an *edf* of 2 indicates a quadratic relationship, and so on. The association between age and rank was statistically significant, approached a sixth-order polynomial function (*edf* = 5.97; Fig. 2), and was consistent with rank trajectories described elsewhere (Foerster et al., 2016). Another finding consistent with the literature was the presence of individual variation in rank trajectory, that is, the chimpanzee ID × age (random effect) term was significant (*edf* = 4.32). Dominance was linearly associated with rank (*edf* = 1.17) such that higher Dominance individuals tended to have higher rank regardless of age (Fig. 3, top). Although the association between Conscientiousness and rank varied across individuals (*edf* = 10.26), the main effect of Conscientiousness was significant (*edf* = 3.69). Individuals with the highest Conscientiousness scores, therefore, tended to have lower rank, while those individuals with Conscientiousness scores that were slightly above average had slightly higher rank (Fig. 3, bottom).

**Figure 4:**
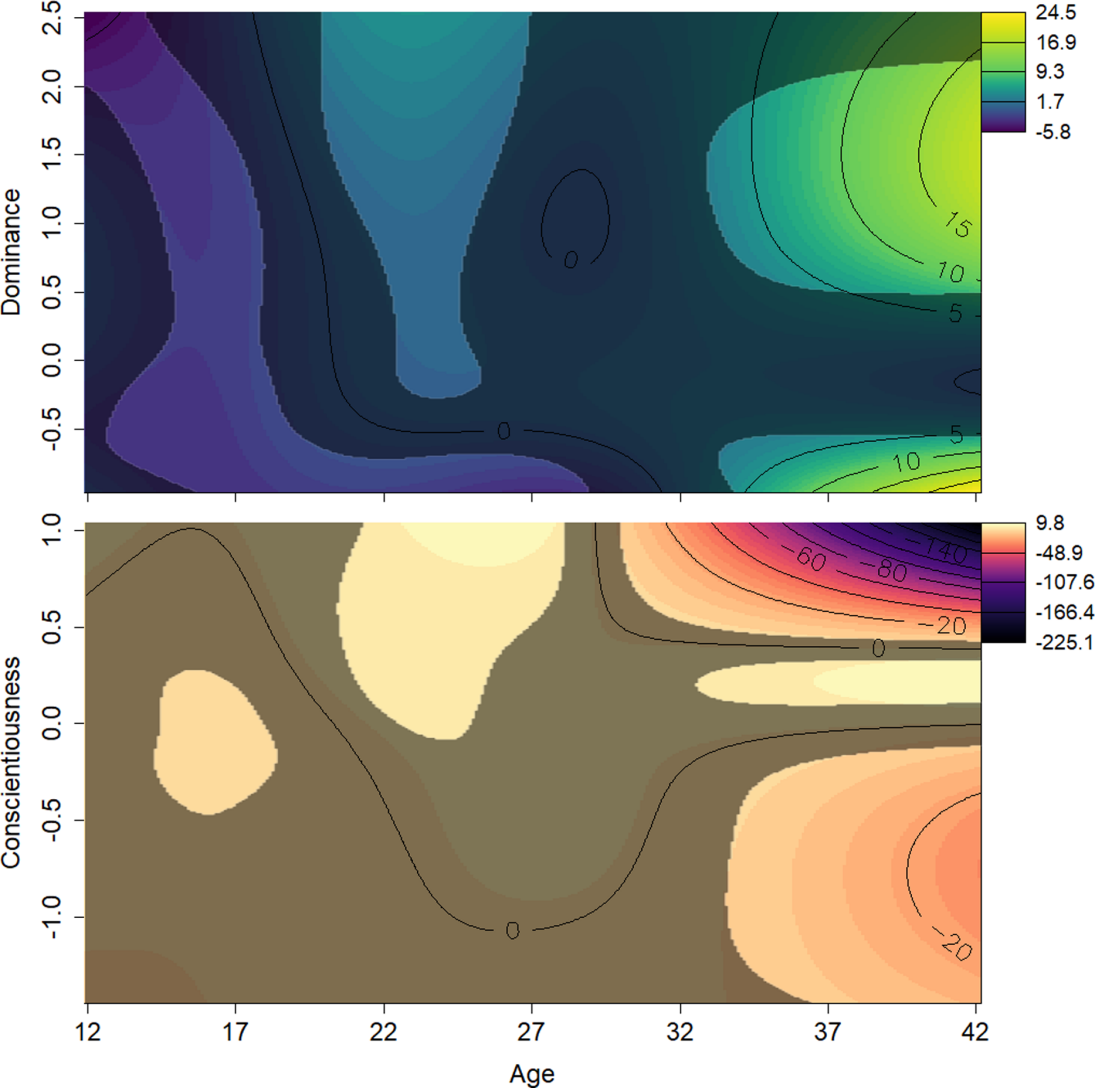
Surfaces illustrating Elo rank score and the interactions between age and personality traits. The surfaces are the result of a generalized additive mixed effects model predicting Elo score from age, scores on all personality traits, and interactions between these terms. The *y* axes show *z*-transformed personality trait scores. Contour lines indicate the magnitude of the association at the intersection of age and personality score. The surface’s darker, less colorful regions represent those areas whose 95% confidence interval includes zero (*i.e.,* are not statistically significant). Light, yellow-green areas represent regions in which age and Dominance values, all else being equal, are associated with higher Elo scores; light orange indicates regions where Conscientiousness and age are associated with higher Elo scores; dark, purple areas represent regions in which age and personality values, both Dominance and Conscientiousness, are associated with lower Elo scores. Males who were either high or low in Dominance tended to be low ranked in early adulthood. Males average or very high in Dominance rose in rank as they entered their prime. Males high in Conscientiousness appeared to have higher rank in their prime.

The effects of the interactions between age and Dominance indicated that all males tend to start out at lower ranks. Young adults (aged around 20 to 25 years) who scored average or higher on Dominance had higher rank scores, while males in the same age range who had low scores on Dominance had relatively lower rank scores (Fig. 4, top). Conscientiousness showed only a few age-dependent associations with rank, but they are difficult to interpret (Fig. 4, bottom). Young males (12–18) of average Conscientiousness appear to have slightly higher rank, as did high Conscientiousness males aged about 21–26.

**Table 2:** Generalized additive mixed model results of regression cardinal Elo score on age and personality smooths. Estimated degrees of freedom (*df*) indicate the penalized number of regression terms associated with this smooth. Reference *dfs* indicate the unpenalized, maximum possible terms. Fixed effects (*ti*) are main and interaction effects of tensor product smooths using cubic regression splines with shrinkage. Random effects (s) smooths are parametric terms penalized by a ridge penalty.

**Model term**	**Estimated *df***	**Reference *df***	*χ* ^2^	***p***
ti (Age)	5.970	6	7,696.943	<0.001
ti (Dominance)	1.172	4	330.975	<0.001
ti (Conscientiousness)	3.690	4	322.321	0.658
ti (Extraversion)	0.617	4	79.524	0.098
ti (Agreeableness)	0.001	4	0.000	0.551
ti (Openness)	1.350	4	418.057	0.001
ti (Neuroticism)	0.000	4	0.000	0.639
ti (Age × Dominance)	13.318	16	1,165.512	0.027
ti (Age × Conscientiousness)	15.869	16	2,914.363	0.019
ti (Age × Extraversion)	11.327	16	2,086.150	<0.001
ti (Age × Agreeableness)	14.916	16	3,441.257	0.118
ti (Age × Openness)	14.961	16	1,494.961	0.021
ti (Age × Neuroticism)	3.141	16	140.932	0.051
s (ID)	0.009	27	0.008	0.241
s (ID × Age)	4.323	28	73.749	0.026
s (ID × Dominance	0.000	27	0.000	0.409
s (ID × Conscientiousness)	10.260	27	945.512	<0.001
s (ID × Extraversion)	0.000	27	0.000	0.207
s (ID × Agreeableness)	3.358	27	54.724	0.054
s (ID × Openness)	0.002	27	0.002	0.206
s (ID × Neuroticism)	0.000	27	0.000	0.224

### Study 2: personality and reproductive success

**Figure 5:**
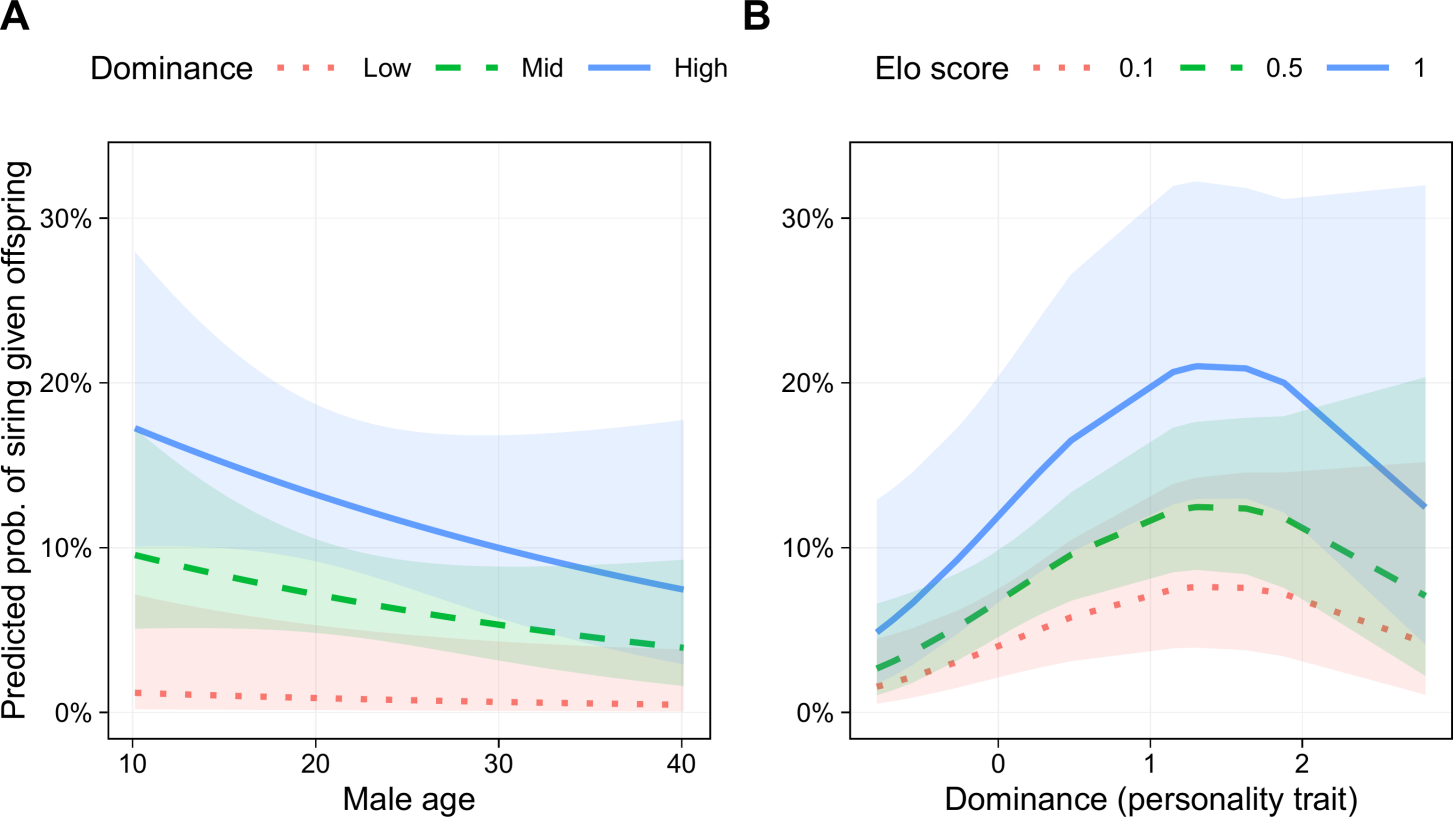
Predicted probability of siring a given offspring based on Dominance, age, and Elo score. (A) Males with higher Dominance scores were most successful at all ages. Low, Mid, and High Dominance scores correspond to values of −1.5, 0, and 1.5, respectively, in *z*-transformed Dominance scores. (B) Males that were most successful had above average (but not the highest) Dominance trait scores. Elo score showed a linear positive association with siring success, with the highest-Elo males (blue line) most successful regardless of Dominance trait score. Results based on 55 siring events, with 24 unique mothers and 22 unique males.

Six models had lower AICc scores than the base model, three of which were within 2 AICc points of the best model, indicating roughly equivalent support (Table 3). In addition to the terms in the base model, the best model (Akaike weight = 0.269) included a linear term for Dominance and a quadratic term for this trait (*i.e.,* Dominance^2^). The parameters for the best fitting model are presented in Table 4. Male age was negatively associated with siring success, although the 95% confidence interval did not exclude one (odds ratio (OR) = 0.78, 95% CI [0.55–1.11], *p* = .171; Fig. 5B). Genetic relatedness was also negatively related to siring success (OR = 0.15, 95% CI [0.03–0.71], *p* = .017). Male Elo score was positively associated with siring success (OR = 1.44, 95% CI [1.05–1.97], *p* = .023; Fig. 5A). Finally, even though this model controlled for rank, Dominance was associated with a greater likelihood of siring offspring (OR = 2.59, 95% CI [1.30–5.16], *p* = .007), and this relationship was not linear (Dominance^2^ OR = 0.72, 95% CI [0.52–1.00], *p* = .047), such that the most successful males were those with above average, but not the highest, Dominance scores (Fig. 5B).

**Table 3:** Model comparison table for models predicting siring success. Base includes male age, male Elo score on the estimated date of siring, and male relatedness to the mother, and random intercepts for male identity. The models are presented in order from the lowest to highest Akaike Information Criterion corrected for small sample sizes (AICc). Lower AICc values indicate a better balance of model fit and model parsimony. *df* refers to the number of degrees of freedom in the model. Δ_*i*_ is the difference between the AICc of the best fitting model and a model, *i*. weight_*i*_ is the probability that a model, *i*, is the best fitting model.

**Model**	***df***	**AICc**	Δ_*i*_	**weight**_*i*_
Base model + Dominance + Dominance^2^	7	357.616	0.000	0.269
Base model + Dominance × Age + Dominance^2^	8	358.392	−0.777	0.182
Base model + Dominance × Age	7	359.292	−1.676	0.116
Base model + Dominance	6	359.649	−2.034	0.097
Base model + Dominance × Age + Dominance^2^× Age	9	360.454	−2.839	0.065
Base model + Dominance × Elo score	7	360.765	−3.150	0.056
Base model	5	361.129	−3.514	0.046
Base model + Conscientiousness × Elo score	7	361.526	−3.910	0.038
Base model + Conscientiousness	6	361.569	−3.953	0.037
Base model + Dominance + Conscientiousness	7	361.658	−4.042	0.036
Base model + All six trait scores	11	363.054	−5.438	0.018
Base model + Conscientiousness × Age	7	363.130	−5.514	0.017
Base model + Conscientiousness + Conscientiousness^2^	7	363.407	−5.792	0.015
Base model + Conscientiousness × Age + Conscientiousness^2^	8	365.073	−7.458	0.006

**Table 4:** Parameters from the best model for predicting siring success.

**Predictor**	**Odds ratio**	**95% Confidence Interval**	***p***
Intercept	0.07	0.05–0.11	<0.001
Male age	0.78	0.55–1.11	0.171
Male Elo score	1.44	1.05–1.97	0.023
Relatedness with mother	0.15	0.03–0.71	0.017
Dominance	2.59	1.30–5.16	0.007
Dominance^2^	0.72	0.52–1.00	0.047

The two best-supported models included Dominance^2^, and models including this term accounted for 52% of total model weight, indicating substantial support for a quadratic relationship between Dominance and siring success. Nevertheless, several models with better fit than the base model contained only linear effects of Dominance (Table 3). Therefore, although we found strong support for a relationship between Dominance and male siring success, and substantial support for this relationship suggests that it is nonlinear, without a larger sample, it is too soon to determine the precise shape of this relationship. The siring success model that included all six personality traits had a poorer fit than the models with only Dominance.

## Discussion

In wild male chimpanzees living in Gombe National Park, higher Dominance and lower Conscientiousness were associated with higher rank scores. Male chimpanzees that rise to the alpha position sire a disproportionate share of offspring (Feldblum et al., 2021), so personality traits that predispose males to higher rank should be expected to increase lifetime reproductive success. Dominance was associated with higher reproductive success, and this association was still significant if models included rank score at the time of siring. In other words, the effect of Dominance was not primarily mediated by rank.

On the other hand, we did not find evidence for age-varying associations between either Dominance or Conscientiousness, and either rank or reproductive success. In sum, although our results support functional implications for personality traits (both traits, and especially Dominance, were associated with rank and reproductive success), adaptive personality variation in the wild male chimpanzees examined in this study did not appear to persist because alternative phenotypes are associated with strategies for reproducing at different times in life (Wolf et al., 2007). Because the GAMMs we used to examine the association between personality and rank were sensitive to parsimonious non-linear relationships, our inability to find evidence for clear trade-offs between early and later life rank attainment is particularly noteworthy.

McElreath et al. (2007) predicted that the model by Wolf et al. (2007) would not hold in long-lived species. This prediction appears to have been borne out by our data. These findings, thus, suggest that some other mechanism or mechanisms are responsible for the persistence of heritable variance in personality traits in this species. One possibility is that another trade-off maintains variance in these traits, such that unexamined costs of high Dominance and low Conscientiousness offset their benefits reported here. One possible cost is risk of mortality (Stamps, 2007; Biro & Stamps, 2008), especially as, in humans, analogous traits are related to greater mortality risk, and poorer health more generally (Strickhouser, Zell & Krizan, 2017). Such trade-offs have been reported in several species, including, for example, bighorn sheep rams (*Ovis canadensis*; Réale et al., 2009) and North American red squirrels (*Tamiasciurus hudsonicus*; Boon, Réale & Boutin, 2008). A second scenario is that variation is maintained by selection in heterogeneous environments, such that the relationship between traits and fitness varies across space and/or over time (Penke, Denissen & Miller, 2007). One prediction of this model is that the association between these traits and fitness would vary across seasons, habitats and/or communities, and across cohorts that differ in factors such as population density. A third possibility is that one or more personality traits are related to phenotypic quality (Parker, 1982), and that early successes or failures in competition with other males could lead to feedback loops (Sih et al., 2004; Luttbeg & Sih, 2010; Sih et al., 2015) that shape behavioral strategies for rank competition. If this is the case, one would predict that Dominance and Conscientiousness are independently associated with measures of phenotypic quality in males, such as body size, and that measures of male quality explain some or all the associations between personality traits and rank attainment.

Although chimpanzees experience age-related changes in social behavior (Rosati et al., 2020; Thompson González et al., 2021) and personality (King, Weiss & Sisco, 2008; Haux et al., 2023) resembling aging patterns in humans, studies in both captive and wild chimpanzees suggests that individual behavioral phenotypes are stable (King, Weiss & Sisco, 2008; Weiss et al., 2017; Tkaczynski et al., 2020; Thompson González et al., 2021). Thus, given the present findings of associations between personality traits and fitness, another productive avenue for future studies would be to identify which behaviors related to Dominance and Conscientiousness are responsible for higher rank and reproductive success in males. Studies of captive chimpanzees suggest that Dominance is related to higher rates of aggressive displays (Pederson, King & Landau, 2005), raising the possibility that the greater siring success of males who are high in Dominance could result from male sexual coercion (*e.g.*, Feldblum et al., 2014).

These present study’s findings complement findings from a recent study in Gombe (Massaro et al., 2022), which investigated the predictors of individual differences in boundary patrol participation. As predicted, males that were higher in Dominance and lower in Conscientiousness were more likely to participate in patrols. However, the sizes of these effects were modest, and model-averaged confidence intervals included zero for both traits. This may be because rates of participation were quite high for all males as males likely benefit from patrol participation in a number of ways (see Massaro et al., 2022 for a discussion). The present findings highlight the need for additional work that links personality traits with inter-individual behavioral differences in the wild.

Our findings also highlight the importance of observer ratings for capturing patterns of behaviors that may not always be recorded by standard ethological measures, but which may nevertheless form people’s impressions of individual animals. For instance, Dominance does not describe specific behaviors per se, but reflects a continuum ranging from being assertive and forceful in social interactions, fearlessness, incautiousness, and more intelligent to being hesitant and deferential, fearful, cautious, and less intelligent. Capturing this variation would be difficult using only standard measures such as rank and rates of aggression. Moreover, as we demonstrated in this study, the use of ratings makes it possible to take advantage of entire datasets, including in the present case where there are gaps in behavioral data during early parts of the study period.

Finally, our findings speak to the claim that ratings also produce biologically meaningful personality data (Weiss & Adams, 2013). Dominance and Conscientiousness had similar associations with reproductive success as boldness and aggression (Smith & Blumstein, 2008). Thus, these rating-based traits appear to be measures of the same constructs as ecologically relevant traits measured using behavioral tests, codings, and direct observations.

This study is not without limitations. For one, although repeated measures analyses, which maximized statistical power (Guo et al., 2013) were used, the sample comprised a small number of chimpanzees that belonged to a single community. The present findings might therefore not generalize to other communities. Replicating and extending these findings in large, multisite studies of chimpanzees (*e.g.*, Wilson et al., 2014), and to populations of other species, including humans, is necessary if we are to further understand the ultimate origins of personality in long-lived, slowly reproducing species.

## Conclusions

Personality varies for reasons that remain poorly understood. Longitudinal studies across taxa are needed to clarify how this variation relates to fitness. Our findings add to the literature documenting fitness implications of animal personality traits, and the literature addressing whether personality ratings provide biologically meaningful data, but they do not support the persistence of variation in these traits *via* trade-offs in the timing of reproduction. Instead, our findings suggest that among male chimpanzees, the fitness-related benefits of high Dominance, and to a lesser extent low Conscientiousness, persist throughout the life course. This finding clears the way for tests of additional theoretical mechanisms that may account for the persistence of variation in animal personality traits, but which require studies of multiple communities of chimpanzees, and of other long-lived species, including our own.

##  Supplemental Information

10.7717/peerj.15083/supp-1 Text S1 Supplementary methods

10.7717/peerj.15083/supp-2 Table S1 Generalized additive mixed model results of regression cardinal Elo score on age and personality smooths, with added random effect for date Estimated degrees of freedom (*df*) indicate the penalized number of regression terms associated with this smooth. Reference *df* indicate the unpenalized, maximum possible terms. Fixed effects (ti) are main and interaction effects of tensor product smooths using cubic regression splines with shrinkage. Random effects (s) smooths are parametric terms penalized by a ridge penalty.

10.7717/peerj.15083/supp-3 Table S2 Cross-validation statistics for the main full generalized additive mixed model CV = cross validation, MSE = Mean squared error of the model predicted values versus the real values. Test values represent the score of the model predicting the values it was fitted with. Forward chaining CV values represent the scores from models fitted to temporally sequential incremental data. See the supplementary methods for more on this approach.

10.7717/peerj.15083/supp-4 Table S3 Cross-validation statistics for the generalized additive mixed model with age, personality, their interactions, and random effects for date CV = cross validation, MSE = Mean squared error of the model predicted values versus the real values. Test values represent the score of the model predicting the values it was fitted with. Forward chaining CV values represent the scores from models fitted to temporally sequential incremental data. See the supplementary methods for more on this approach.

10.7717/peerj.15083/supp-5 Supplemental Information 1 Supplementary figures

10.7717/peerj.15083/supp-6 Supplemental Information 2 Data and code used to conduct preliminary validity check

10.7717/peerj.15083/supp-7 Supplemental Information 3 Data and code for rank analysis (GAMM and cross-validation)

10.7717/peerj.15083/supp-8 Supplemental Information 4 Data and code used to conduct analysis of reproductive success

## Additional Information and Declarations

### Field Study Permissions

The following information was supplied relating to field study approvals (*i.e.*, approving body and any reference numbers): Data collection in Gombe National Park was approved by the Tanzania National Parks.

### Data Availability

The following information was supplied regarding data availability: The R data files and code used to analyze the data and produce the results are available in the Supplemental Files.
