# Supplementary material for: Personality traits, rank attainment, and siring success throughout the lives of male chimpanzees of Gombe National Park
Source: PeerJ. 2023 Apr 24;11:e15083. doi: 10.7717/peerj.15083 (PMC10135409; doi:10.7717/peerj.15083)
Supplement: Text S1 [file peerj-11-15083-s001.docx]

### Supplementary methods

### Inclusion of study subjects

Study subjects were male eastern chimpanzees from the Kasekela community in Gombe National Park, Tanzania. For the first analysis of personality and dominance rank, we included all males for whom we had personality and rank data between 1978 and 2015. Personality ratings data were available for 46 males in this community [(Weiss et al., 2017)](https://www.zotero.org/google-docs/?l9zJv1). Our method of assigning ranks (see Methods) requires a minimum of one 'win' and one 'loss' from each individual [(Foerster et al., 2016)](https://www.zotero.org/google-docs/?tHGPxJ). For three males, we lacked sufficient data to meet this criterion. We also excluded five individuals who did not survive to age 12, and 10 individuals who died before 1978, the earliest year for which we have dominance rank data (see Methods).

The remaining 28 males comprising our study sample were present for a mean of 14.1 years beyond the age of 12 (range 1.7 to 30.7 years). To test whether excluding the 18 males biased our sample, that is, whether they differed in their personalities, we compared the six personality scores of the 28 subjects to those of the 18 males that were excluded. The two groups did not differ on any of the six traits (see below).

**Comparison of chimpanzees that were (n = 28) and were not (n = 18) included as subjects using Welch’s *t*-tests.**

Means for personality traits in each group expressed as *z*-scores (mean = 0; sd = 1). Tests were two-sided and no effort was made to control for multiple tests.

| **Trait** | **Excluded** | **Included** | **t** | **df** | ***p*** |
| --- | --- | --- | --- | --- | --- |
| Dominance | -0.28 | 0.18 | -1.514 | 34.512 | 0.1391 |
| Extraversion | -0.25 | 0.16 | -1.362 | 36.511 | 0.1815 |
| Conscientiousness | 0.17 | -0.11 | 0.969 | 39.656 | 0.3383 |
| Agreeableness | -0.06 | 0.04 | -0.346 | 35.572 | 0.7314 |
| Neuroticism | -0.27 | 0.17 | -1.498 | 36.248 | 0.1427 |
| Openness | 0.04 | -0.03 | 0.204 | 31.702 | 0.8398 |

For the second analysis of personality and reproductive success, we began with a set of 60 siring events with known paternity between 1986 and 2014, with 25 unique mothers. We analyzed data by siring event, using personality and other individual traits to predict likelihood of male siring success. We included males as potential sires if they were at least 11 years old on the siring date, as the youngest male known to sire an offspring in Gombe was 11.4 years old [(Wroblewski et al., 2009)](https://www.zotero.org/google-docs/?oAZA6e). This led to an initial dataset containing 33 unique males. We excluded four siring events because we were missing genetic relatedness data for at least four potential sires on the date of siring, and we further excluded one male for whom we were missing genetic relatedness data and so did not know how related they were with all females (see Methods). We also excluded one siring event for which the sire did not have personality ratings, and we excluded two males from other siring events for which we were missing personality ratings. Finally, we excluded two males that were suspected of being sterile from the analysis (Pax, who received severe testicular injuries before puberty, and Goblin after 1989, when he received severe testicular injuries in a fight which were believed to render him sterile; [Goodall, 1986, 1992)](https://www.zotero.org/google-docs/?0OFoVA). This resulted in a data set for the siring analysis of 55 siring events, with 24 unique mothers and 22 unique males. The dataset had a median of 11 (range 8 to 14) potential sires per siring event.

### Personality trait calculations

Trait scores were calculated using the following formulas (1):

Dominance = (dominant - dependent.follower + decisive + 8)/3

Extraversion = (−solitary + sociable - individualistic + active + 16)/3

Conscientiousness = (−impulsive - reckless + predictable + 16)/3

Agreeableness= (sympathetic + helpful + sensitive)/3

Neuroticism = (−stable + excitable + 8)/2

Openness = (inventive + inquisitive + innovative)/4

### Validation analyses

#### Methods

Cross validation is the gold standard for verifying predictive model fit [(Browne, 2000)](https://www.zotero.org/google-docs/?3BDNCW), particularly with machine learning methods such as generalized additive models. We calculated *K*-fold cross validation scores in stratified sample folds. That is, in each of *K*=10 folds we balanced the number of observations from each chimpanzee in the dataset. The total cross validation score is the average of the mean squared errors of the models’ predicted values and the real values for each combination of folds.

*k*-fold cross validation was not ideally suited to evaluate our models due to high temporal autocorrelation between measurements. In other words, because rank, and thus Elo score, is relatively stable across time, each observation will be similar to the ones preceding and following it. In balanced folds, this will inflate the predictive power of the model. A better way to evaluate the predictive power of models such as ours is to treat them as the time series they are and to cross validate these models using forward chaining, as is normally done in time series models. In forward chaining, the dataset is split into folds, but each fold is temporally sequential: fold 2 follows fold 1, fold 3 follows fold 2, and so on. Folds are grouped sequentially, the model is fitted to those data, and then the model is used to predict the values in the following fold. The model is fitted to fold 1 and then the mean squared predictive error is calculated with the data in fold 2. Then, the model is fitted to folds 1 and 2, and the mean squared error is calculated with fold 3. This process is repeated until the data have been fitted with *K* folds, so to have the same number of scores to average as with traditional *K-*fold cross validation, *K*+1 folds must be created from the data.

We thus created 11 folds; within each fold, we stratified both temporally and by individual. So, fold 1 contained the first 1/11th of observations from each individual, fold 2 contained the second 1/11th of observations from each individual, and so on. This maintained balance over the folds, which was crucial due to the differing time courses of different individuals’ rank trajectories while they were a member of the group. Folds could then be combined as described above to create temporally consistent subsets of the full dataset.

## References

[Browne MW. 2000. Cross-Validation Methods. *Journal of Mathematical Psychology* 44:108–132. DOI: 10.1006/jmps.1999.1279.](https://www.zotero.org/google-docs/?6UOVa3)

[Foerster S, Franz M, Murray CM, Gilby IC, Feldblum JT, Walker KK, Pusey AE. 2016. Chimpanzee females queue but males compete for social status. *Scientific Reports* 6:1–11. DOI: 10.1038/srep35404.](https://www.zotero.org/google-docs/?6UOVa3)

[Goodall J. 1986. *The Chimpanzees of Gombe: Patterns of Behavior*. Cambridge, Massachusetts: Belknap Press of Harvard University Press.](https://www.zotero.org/google-docs/?6UOVa3)

[Goodall J. 1992. Unusual violence in the overthrow of an alpha male chimpanzee at Gombe. In: *Topics in primatology; Vol. 1 Human Origins*. University of Tokyo Press, 131–142.](https://www.zotero.org/google-docs/?6UOVa3)

[Weiss A, Wilson ML, Collins DA, Mjungu D, Kamenya S, Foerster S, Pusey AE. 2017. Personality in the chimpanzees of Gombe National Park. *Scientific Data*:1–18. DOI: 10.1038/sdata.2017.146.](https://www.zotero.org/google-docs/?6UOVa3)

[Wroblewski EE, Murray CM, Keele BF, Schumacher-Stankey JC, Hahn BH, Pusey AE. 2009. Male dominance rank and reproductive success in chimpanzees, Pan troglodytes schweinfurthii. *Animal Behaviour* 77:873–885. DOI: 10.1016/j.anbehav.2008.12.014.](https://www.zotero.org/google-docs/?6UOVa3)
