# Supplementary material for: Personality traits, rank attainment, and siring success throughout the lives of male chimpanzees of Gombe National Park
Source: PeerJ. 2023 Apr 24;11:e15083. doi: 10.7717/peerj.15083 (PMC10135409; doi:10.7717/peerj.15083)
Supplement: Table S1 — Estimated degrees of freedom (df) indicate the penalized number of regression terms associated with this smooth. Reference df indicate the unpenalized, maximum possible terms. Fixed effects (ti) are main and interaction effects of tensor product smooths using cubic regression splines with shrinkage. Random effects (s) smooths are parametric terms penalized by a ridge penalty. [file peerj-11-15083-s002.docx]

| **Model term** | **Estimated *df*** | **Reference *df*** | **𝛘^2^** | ***p*** |
| --- | --- | --- | --- | --- |
| ti(Age) | 5.964 | 6 | 6182.264 | < 0.001 |
| ti(Dominance) | 1.058 | 4 | 1354.369 | 0.008 |
| ti(Conscientiousness) | 3.795 | 4 | 3061.741 | 0.005 |
| ti(Extraversion) | 0.001 | 4 | 0.000 | 0.218 |
| ti(Agreeableness) | 0.001 | 4 | 0.000 | 0.883 |
| ti(Openness) | 0.000 | 4 | 0.000 | 0.367 |
| ti(Neuroticism) | 0.001 | 4 | 0.002 | 0.122 |
| ti(Age × Dominance) | 11.27 | 16 | 793.504 | 0.210 |
| ti(Age × Conscientiousness) | 15.86 | 16 | 3404.718 | 0.085 |
| ti(Age × Extraversion) | 11.31 | 16 | 1325.363 | 0.026 |
| ti(Age × Agreeableness) | 5.795 | 16 | 392.583 | 0.065 |
| ti(Age × Openness) | 14.12 | 16 | 5229.720 | 0.0390 |
| ti(Age × Neuroticism) | 12.10 | 16 | 1990.359 | 0.030 |
| s(Date) | 8.587 | 9 | 1729.729 | < 0.001 |
| s(ID) | 0.000 | 27 | 0.001 | 0.560 |
| s(ID × Age) | 8.120 | 28 | 389.807 | < 0.001 |
| s(ID × Dominance | 0.000 | 27 | 0.000 | 0.850 |
| s(ID × Conscientiousness) | 0.003 | 27 | 0.003 | 0.445 |
| s(ID × Extraversion) | 0.000 | 27 | 0.000 | 0.408 |
| s(ID × Agreeableness) | 0.000 | 27 | 0.000 | 0.502 |
| s(ID × Openness) | 6.088 | 27 | 138.912 | 0.568 |
| s(ID × Neuroticism) | 6.499 | 27 | 699.092 | 0.031 |
