# Supplementary material for: Personality traits, rank attainment, and siring success throughout the lives of male chimpanzees of Gombe National Park
Source: PeerJ. 2023 Apr 24;11:e15083. doi: 10.7717/peerj.15083 (PMC10135409; doi:10.7717/peerj.15083)
Supplement: Table S3 — CV = cross validation, MSE = Mean squared error of the model predicted values versus the real values. Test values represent the score of the model predicting the values it was fitted with. Forward chaining CV values represent the scores from models fitted to temporally sequential incremental data. See the supplementary methods for more on this approach. [file peerj-11-15083-s004.docx]

| **Score type** | **MSE** |
| --- | --- |
| Test | 0.0426 |
| 10-fold CV average | 0.0075 |
| **Fold(s)** | **Forward chaining CV** |
| 1 | 0.0572 |
| 1 and 2 | 0.0246 |
| 1 through 3 | 0.0271 |
| 1 through 4 | 0.0382 |
| 1 through 5 | 0.0382 |
| 1 through 6 | 0.0840 |
| 1 through 7 | 0.0594 |
| 1 through 8 | 0.0521 |
| 1 through 9 | 0.0392 |
| 1 through 10 | 0.0322 |
| Chaining CV average | 0.0452 |
