# Supplementary material for: Personality traits, rank attainment, and siring success throughout the lives of male chimpanzees of Gombe National Park
Source: PeerJ. 2023 Apr 24;11:e15083. doi: 10.7717/peerj.15083 (PMC10135409; doi:10.7717/peerj.15083)
Supplement: Supplemental Information 1 [file peerj-11-15083-s005.docx]

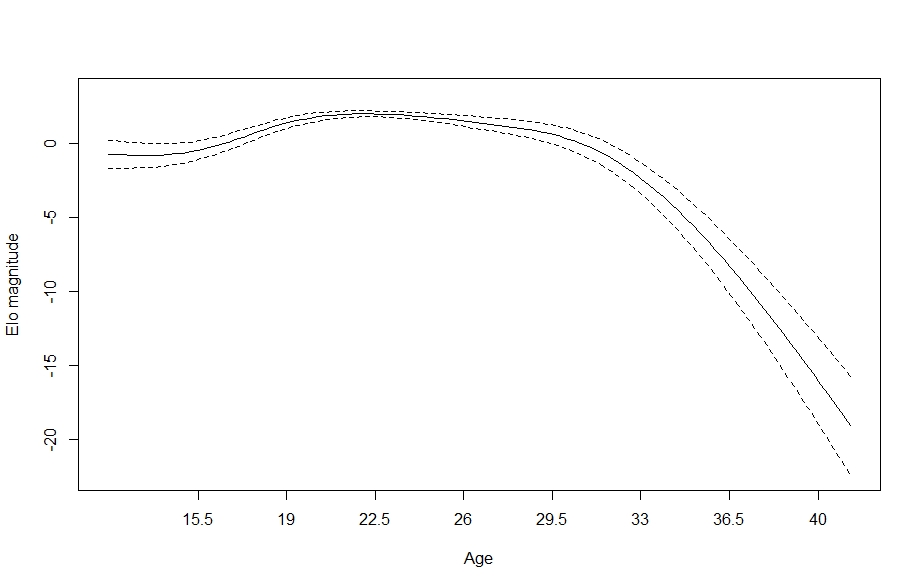


**Figure S1. Regression spline of Elo rank score associated with different ages, in the most complex GAM model with a random effect of date.** The solid line indicates the spline and the dashed lines indicate the boundaries of the 95% confidence region (± 2

standard errors).


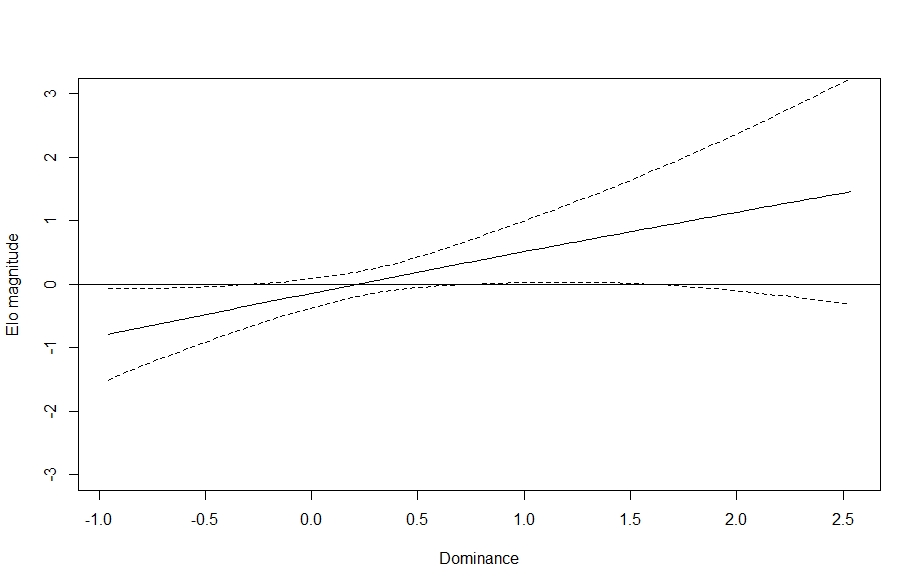


**Figure S2. Regression splines of Elo rank score associated with Dominance, in the most complex GAM model with a random effect of date.** The solid black line indicates the spline, dashed lines indicate the bounds of the 95% confidence region (± 2 standard errors). The x-axis is in z-scored units of Dominance.


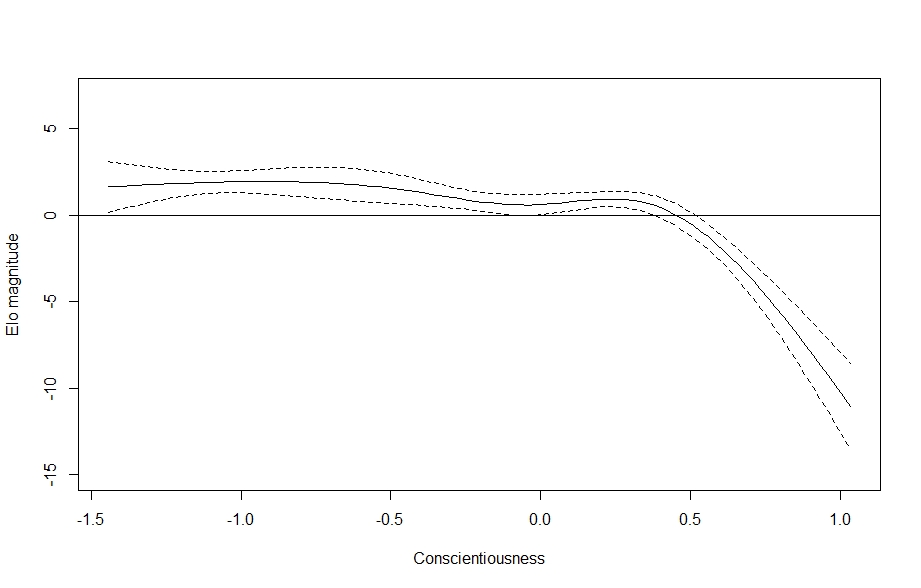


**Figure S3. Regression splines of Elo rank score associated with Conscientiousness, in the most complex GAM model with a random effect of date.** The solid black line indicates the spline, dashed lines indicate the bounds of the 95% confidence region (± 2 standard errors). The x-axis is in z-scored units of Conscientiousness.


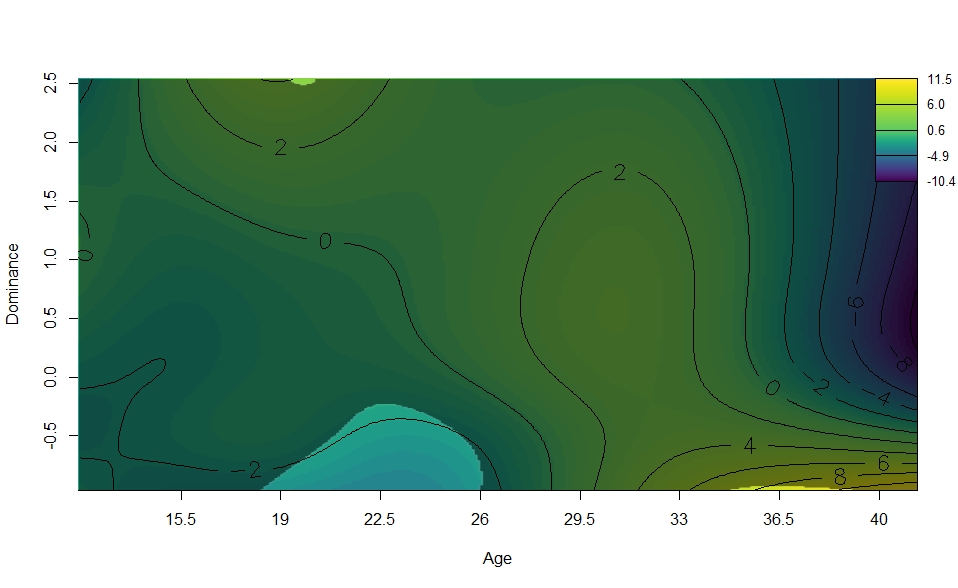


**Figure S4. Surface illustrating Elo rank score and the interactions between age and Dominance, in the most complex GAM model with a random effect of date.** The surface is the result of a GAM model predicting Elo score from age, scores on all personality traits, and interactions between these terms. Contour lines indicate the magnitude of the association at the intersection of age and Dominance, which is z-scored. The surface’s shaded, less colorful regions represent those areas whose 95% confidence interval includes zero (i.e., are not statistically significant).


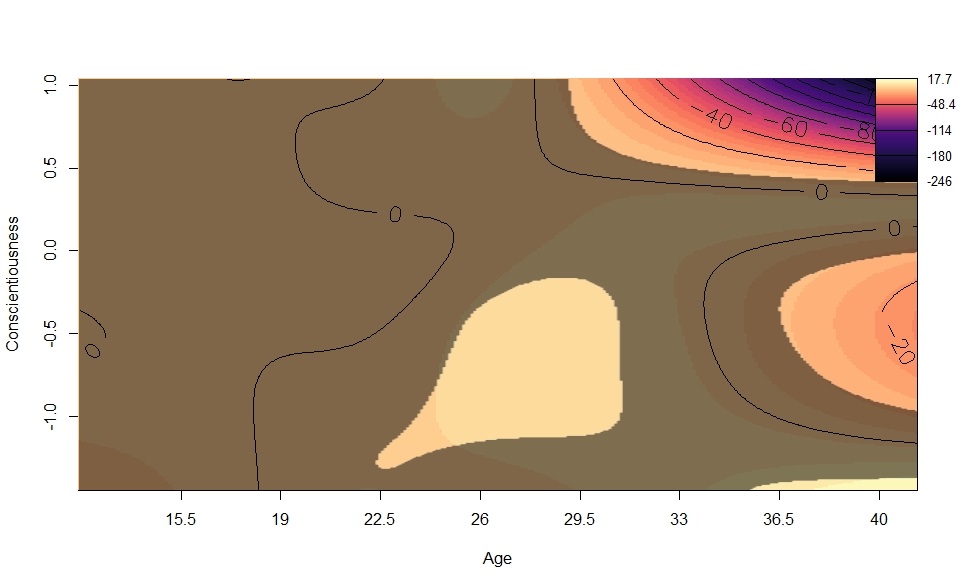


**Figure S5. Surface illustrating Elo rank score and the interactions between age and Conscientiousness, in the most complex GAM model with a random effect of date.** The surface is the result of a GAM model predicting Elo score from age, scores on all personality traits, and interactions between these terms. Contour lines indicate the magnitude of the association at the intersection of age and Conscientiousness, which is z-scored. The surface’s shaded, less colorful regions represent those areas whose 95% confidence interval includes zero (i.e., are not statistically significant).
